# 3-Iodo-1*H*-pyrazolo­[3,4-*b*]pyridine

**DOI:** 10.1107/S1600536814010009

**Published:** 2014-05-10

**Authors:** Ping-Hsin Huang, Yuh-Sheng Wen, Jiun-Yi Shen

**Affiliations:** a Cardinal Tien College of Healthcare & Management, Taipei, 231, Taiwan; b Institute of Chemistry, Academia Sinica, Nankang, Taipei, Taiwan; c Department of Chemistry, National Taiwan University, Taipei, Taiwan

## Abstract

The title compound, C_6_H_4_IN_3_, is essentially planar, with a dihedral angle of 0.82 (3)° between the planes of the pyridine and pyrazole rings. In the crystal, pairs of mol­ecules are connected into inversion dimers through N—H⋯N hydrogen bonds. C—I⋯N halogen bonds link the dimers into zigzag chains parallel to the *b*-axis direction. The packing also features π–π stacking inter­actions along (110) with inter­planar distances of 3.292 (1) and 3.343 (1) Å, and centroid–centroid distances of 3.308 (1) and 3.430 (1) Å.

## Related literature   

For the production of anti­tumor agents, see: Huang *et al.* (2007 ▶); Ye *et al.* (2009 ▶). For a related structure, see: Huang *et al.* (2013 ▶).
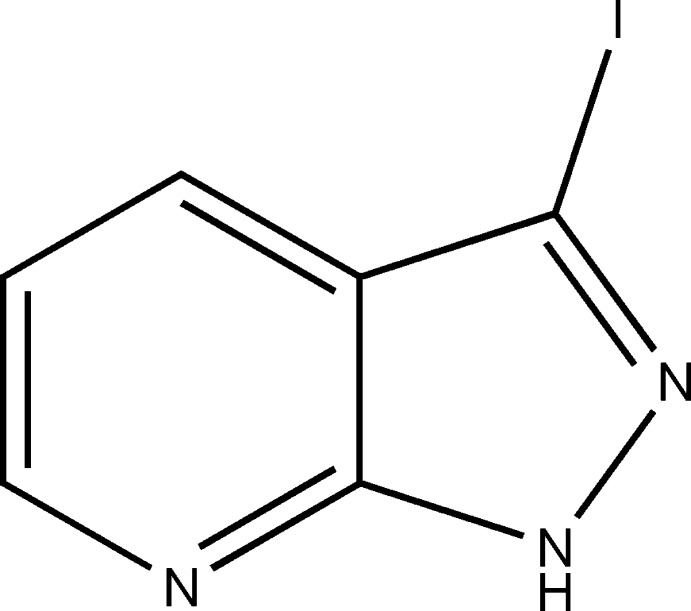


## Experimental   

### 

#### Crystal data  

C_6_H_4_IN_3_
*M* *_r_* = 245.02
Monoclinic, 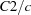

*a* = 10.7999 (13) Å
*b* = 7.7939 (9) Å
*c* = 17.406 (2) Å
β = 101.748 (2)°
*V* = 1434.5 (3) Å^3^
*Z* = 8
Mo *K*α radiation
μ = 4.38 mm^−1^
*T* = 150 K
0.35 × 0.32 × 0.25 mm

#### Data collection  

Bruker SMART APEX CCD area-detector diffractometer
Absorption correction: multi-scan (*SADABS*; Bruker, 1996 ▶) *T*_min_ = 0.309, *T*_max_ = 0.407
5315 measured reflections
1470 independent reflections
1423 reflections with *I* > 2σ(*I*)
*R*_int_ = 0.022

#### Refinement  

*R*[*F* ^2^ > 2σ(*F* ^2^)] = 0.017
*wR*(*F* ^2^) = 0.040
*S* = 1.13
1470 reflections
91 parameters
26 restraints
H-atom parameters constrained
Δρ_max_ = 0.40 e Å^−3^
Δρ_min_ = −0.46 e Å^−3^

### 

Data collection: *SMART* (Bruker, 2001 ▶); cell refinement: *SAINT* (Bruker, 2001 ▶); data reduction: *SAINT* (Bruker, 2001 ▶; program(s) used to solve structure: *SHELXS97* (Sheldrick, 2008 ▶); program(s) used to refine structure: *SHELXL2013* (Sheldrick, 2008 ▶); molecular graphics: *ORTEP-3 for Windows* (Farrugia, 2012 ▶); software used to prepare material for publication: *WinGX* (Farrugia, 2012 ▶).

## Supplementary Material

Crystal structure: contains datablock(s) ic14830, I. DOI: 10.1107/S1600536814010009/zl2581sup1.cif

Structure factors: contains datablock(s) I. DOI: 10.1107/S1600536814010009/zl2581Isup2.hkl

Supporting information file. DOI: 10.1107/S1600536814010009/zl2581Isup3.cml

CCDC reference: 1000735

Additional supporting information:  crystallographic information; 3D view; checkCIF report

## Figures and Tables

**Table 1:** Hydrogen-bond geometry (Å, °)

*D*—H⋯*A*	*D*—H	H⋯*A*	*D*⋯*A*	*D*—H⋯*A*
N2—H2*A*⋯N1^i^	0.88	2.09	2.926 (3)	159
C6—I1⋯N3^ii^	2.076 (2)	3.013 (2)	5.056 (3)	166.72 (7)

Symmetry codes: (i) 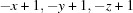; (ii) 
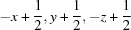
.

**Fig. 1.**
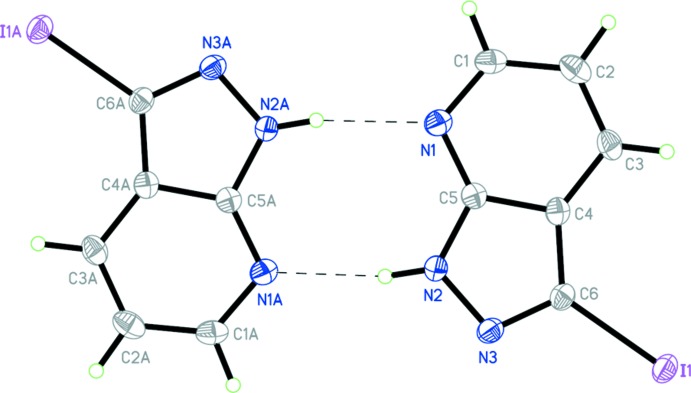
The centrosymmetric dimer molecular structures of the title compound with labeling and displacement ellipsoids drawn at the 30% probability level. H atoms are shown as small spheres of arbitrary radii.

**Fig. 2.**
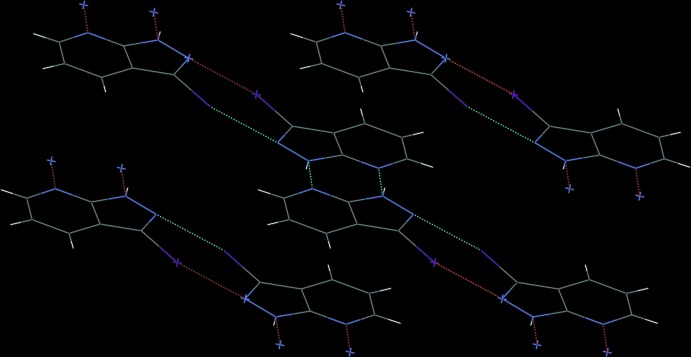
Packing diagram for title compound, viewed along the b axis. H atom have been omitted for clarity.

**Fig. 3.**
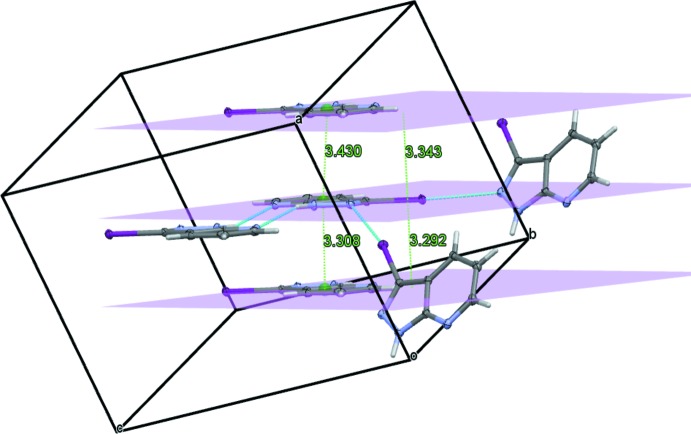
In the crystal, there are significant π···π stacking interactions between molecules.

## supplementary crystallographic information

### 1. Comment

The title compound has been shown to be a precursor for the production of anticancer drugs (Huang *et al.*, 2007; Ye *et al.*, 2009). The molecular structure is shown in Figure 1. The dihedral angle between the pyridine and the pyrazole rings is 0.82 (3)°. (Huang *et al.*, 2013) As shown in Fig.1, N—H···N H-bonds connect molecules into centrosymmetric dimers. The molecules connected the H-bonds are arranged in a parallel but non-coplanar fashion, with the planes of the two molcecules being about 0.67 Å apart. C—I···N halogen bonds create zig zag chains parallel to the *b* axis direction, Fig. 2. Packing is also facilitated through π···π stacking interactions along (1 1 0) with interplanar distances of 3.292 (1) and 3.343 (1) Å, and centroid to centroid distances of 3.308 (1) and 3.430 (1) Å (Fig 3.). Molecules in the crystal structure are thus connected through N—H···N hydrogen bonding interactions, through a C—I···N halogen bond as well as π···π stacking interactions that help to stabilize the crystal structure.

### 2. Experimental

The compound was synthesized by the following procedure (Ye *et al.*, 2009). Iodine (18.7 g, 73.6 mmol) was added to a solution of 1*H*-pyrazolo[3,4-*b*]pyridine (3.5 g, 29.4 mmol) in DMF (50 ml), followed by KOH (6.6 g, 118.0 mmol). The mixture was stirred at room temperature for 2 h. After that, it was poured into brine and extracted with ethyl acetate and the organic extract was washed with brine and aqueous Na_2_SO_4_, dried and concentrated in vacuum. The residue was purified by recrystallization in CH_2_Cl_2_ and hexane to give a white solid (6.3 g, 87.5%). Crystals suitable for X-ray diffraction were grown from a CH_2_Cl_2_ solution layered with hexane at room temperature. ^1^H NMR (CDCl_3_, 400 MHz): 13.18 (br, 1 H), 8.64 (dd, 1 H, J = 4.8, 1.6 Hz), 7.89 (dd, 1 H, J = 8.4, 1.6 Hz), 7.25–7.22 (m, 1H). Mass spectrum: *m/e*245 (*M*^+^), calcd. (245.02).

### 3. Refinement

H atoms were located in difference map but were positioned with idealized geometry and refined isotropic with *U*_iso_(H) = 1.2*U*_eq_(C,*N*).

### Crystal data

**Table:**

C_6_H_4_IN_3_	*F*(000) = 912
*M**_r_* = 245.02	*D*_x_ = 2.269 Mg m^−^^3^*D*_m_ = 2.269 Mg m^−^^3^*D*_m_ measured by not measured
Monoclinic, *C*2/*c*	Mo *K*α radiation, λ = 0.71073 Å
*a* = 10.7999 (13) Å	Cell parameters from 4550 reflections
*b* = 7.7939 (9) Å	θ = 2.4–27.5°
*c* = 17.406 (2) Å	µ = 4.38 mm^−^^1^
β = 101.748 (2)°	*T* = 150 K
*V* = 1434.5 (3) Å^3^	Block, colorless
*Z* = 8	0.35 × 0.32 × 0.25 mm

### Data collection

**Table:**

Bruker SMART APEX CCD area-detector diffractometer	1470 independent reflections
Radiation source: fine-focus sealed tube	1423 reflections with *I* > 2σ(*I*)
Graphite monochromator	*R*_int_ = 0.022
ω scans	θ_max_ = 26.4°, θ_min_ = 2.4°
Absorption correction: multi-scan (*SADABS*; Bruker, 1996)	*h* = −14→13
*T*_min_ = 0.309, *T*_max_ = 0.407	*k* = −10→9
5315 measured reflections	*l* = −22→22

### Refinement

**Table:**

Refinement on *F*^2^	Primary atom site location: structure-invariant direct methods
Least-squares matrix: full	Secondary atom site location: difference Fourier map
*R*[*F*^2^ > 2σ(*F*^2^)] = 0.017	Hydrogen site location: inferred from neighbouring sites
*wR*(*F*^2^) = 0.040	H-atom parameters constrained
*S* = 1.13	*w* = 1/[σ^2^(*F*_o_^2^) + (0.0156*P*)^2^ + 1.9173*P*] where *P* = (*F*_o_^2^ + 2*F*_c_^2^)/3
1470 reflections	(Δ/σ)_max_ = 0.003
91 parameters	Δρ_max_ = 0.40 e Å^−^^3^
26 restraints	Δρ_min_ = −0.46 e Å^−^^3^

### Special details

**Table:**

Geometry. All e.s.d.'s (except the e.s.d. in the dihedral angle between two l.s. planes) are estimated using the full covariance matrix. The cell e.s.d.'s are taken into account individually in the estimation of e.s.d.'s in distances, angles and torsion angles; correlations between e.s.d.'s in cell parameters are only used when they are defined by crystal symmetry. An approximate (isotropic) treatment of cell e.s.d.'s is used for estimating e.s.d.'s involving l.s. planes.
Refinement. Refinement of *F*^2^ against ALL reflections. The weighted *R*-factor *wR* and goodness of fit *S* are based on *F*^2^, conventional *R*-factors *R* are based on *F*, with *F* set to zero for negative *F*^2^. The threshold expression of *F*^2^ > σ(*F*^2^) is used only for calculating *R*-factors(gt) *etc*. and is not relevant to the choice of reflections for refinement. *R*-factors based on *F*^2^ are statistically about twice as large as those based on *F*, and *R*-factors based on ALL data will be even larger.

### Fractional atomic coordinates and isotropic or equivalent isotropic displacement parameters (Å^2^)

**Table:**

	*x*	*y*	*z*	*U*_iso_*/*U*_eq_	
I1	0.23044 (2)	1.04283 (2)	0.29263 (2)	0.02252 (7)	
N1	0.46725 (18)	0.6969 (3)	0.56007 (11)	0.0202 (4)	
N2	0.44327 (18)	0.6579 (3)	0.42063 (11)	0.0196 (4)	
H2A	0.4869	0.5622	0.4219	0.023*	
N3	0.38503 (19)	0.7396 (3)	0.35341 (11)	0.0201 (4)	
C1	0.4368 (2)	0.8083 (3)	0.61183 (13)	0.0226 (5)	
H1	0.4641	0.7822	0.6660	0.027*	
C2	0.3682 (2)	0.9594 (3)	0.59272 (15)	0.0249 (5)	
H2	0.3517	1.0325	0.6332	0.030*	
C3	0.3242 (2)	1.0027 (3)	0.51533 (15)	0.0223 (5)	
H3	0.2758	1.1038	0.5010	0.027*	
C4	0.3539 (2)	0.8915 (3)	0.45868 (13)	0.0177 (4)	
C5	0.4249 (2)	0.7441 (3)	0.48502 (13)	0.0178 (4)	
C6	0.3326 (2)	0.8785 (3)	0.37596 (13)	0.0189 (4)	

### Atomic displacement parameters (Å^2^)

**Table:**

	*U*^11^	*U*^22^	*U*^33^	*U*^12^	*U*^13^	*U*^23^
I1	0.02567 (11)	0.01896 (10)	0.02189 (10)	0.00157 (6)	0.00242 (7)	0.00504 (6)
N1	0.0215 (10)	0.0196 (10)	0.0186 (9)	−0.0028 (8)	0.0020 (8)	0.0012 (8)
N2	0.0222 (10)	0.0173 (10)	0.0184 (9)	0.0042 (8)	0.0020 (8)	0.0004 (7)
N3	0.0229 (10)	0.0192 (10)	0.0174 (9)	−0.0007 (8)	0.0022 (8)	0.0019 (8)
C1	0.0264 (13)	0.0240 (12)	0.0174 (10)	−0.0075 (10)	0.0043 (9)	−0.0015 (9)
C2	0.0281 (14)	0.0234 (13)	0.0252 (12)	−0.0062 (10)	0.0105 (10)	−0.0064 (10)
C3	0.0241 (13)	0.0159 (11)	0.0287 (12)	−0.0020 (10)	0.0097 (10)	−0.0022 (9)
C4	0.0181 (11)	0.0138 (11)	0.0218 (11)	−0.0036 (9)	0.0051 (9)	0.0011 (9)
C5	0.0171 (11)	0.0160 (11)	0.0200 (11)	−0.0037 (9)	0.0031 (8)	−0.0009 (8)
C6	0.0187 (11)	0.0168 (11)	0.0205 (11)	−0.0006 (9)	0.0023 (9)	0.0026 (9)

### Geometric parameters (Å, º)

**Table:**

I1—C6	2.076 (2)	C1—H1	0.9500
N1—C1	1.339 (3)	C2—C3	1.376 (4)
N1—C5	1.345 (3)	C2—H2	0.9500
N2—C5	1.356 (3)	C3—C4	1.398 (3)
N2—N3	1.368 (3)	C3—H3	0.9500
N2—H2A	0.8800	C4—C5	1.405 (3)
N3—C6	1.318 (3)	C4—C6	1.415 (3)
C1—C2	1.396 (4)		
			
C1—N1—C5	113.2 (2)	C2—C3—H3	121.5
C5—N2—N3	110.93 (18)	C4—C3—H3	121.5
C5—N2—H2A	124.5	C3—C4—C5	117.7 (2)
N3—N2—H2A	124.5	C3—C4—C6	138.5 (2)
C6—N3—N2	106.15 (18)	C5—C4—C6	103.79 (19)
N1—C1—C2	125.3 (2)	N1—C5—N2	126.1 (2)
N1—C1—H1	117.4	N1—C5—C4	126.6 (2)
C2—C1—H1	117.4	N2—C5—C4	107.31 (19)
C3—C2—C1	120.1 (2)	N3—C6—C4	111.8 (2)
C3—C2—H2	120.0	N3—C6—I1	119.88 (16)
C1—C2—H2	120.0	C4—C6—I1	128.30 (17)
C2—C3—C4	117.1 (2)		

### Hydrogen-bond geometry (Å, º)

**Table:**

*D*—H···*A*	*D*—H	H···*A*	*D*···*A*	*D*—H···*A*
N2—H2*A*···N1^i^	0.88	2.09	2.926 (3)	159
C6—I1···N3^ii^	2.08 (1)	3.01 (1)	5.056 (3)	167 (1)

Symmetry codes: (i) −*x*+1, −*y*+1, −*z*+1; (ii) −*x*+1/2, *y*+1/2, −*z*+1/2.
